# Supplementary material for: A New Kv1.3 Channel Blocker from the Venom of the Ant Tetramorium bicarinatum
Source: Toxins (Basel). 2025 Jul 30;17(8):379. doi: 10.3390/toxins17080379 (PMC12389897; doi:10.3390/toxins17080379)
Supplement: Supplementary file 1 [file toxins-17-00379-s001.zip › toxins-3728793-supplementary.pdf]

Supplementary Materials: A New Kv1.3 Channel Blocker from the Venom of the Ant *Tetramorium bicarinatum*

Guillaume Boy, Laurence Jouvensal, Nathan Téné, Jean-Luc Carayon, Elsa Bonnafé, Françoise Paquet, Michel Treilhou, Karine Loth and Arnaud Billet

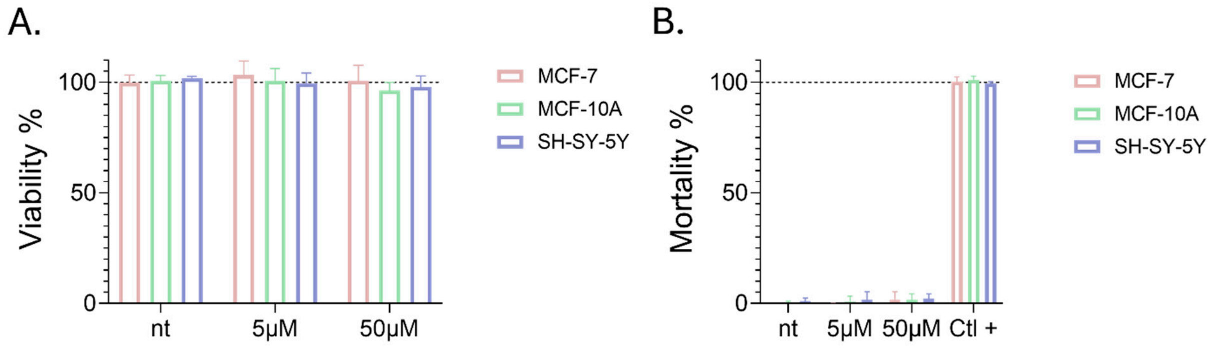

Figure S1: Evaluation of Tb11a cytotoxicity on various human cell lines. MCF-7 (pink), MCF-10A (green) and SH-SY-5Y (purple) were treated with 5 µM or 50 µM Tb11a for 24 hours. Cell viability (A.) was normalized to positive control (non-treated, nt). Cell mortality (B.) was normalized to positive control (Ctl +, lysis buffer). Error bars represent SEM.

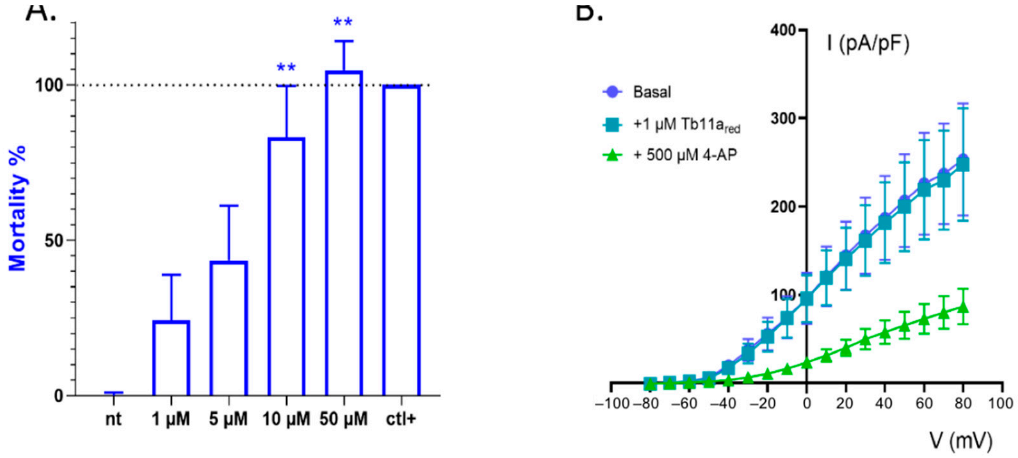

Figure S2: Evaluation of reduced Tb11a cytotoxicity on HEK cells. (A) LDH assay: HEK293T were either non-treated (nt) or treated with increasing reduced Tb11a concentrations (from 1 µM to 50 µM). Cell mortality was normalized to positive control (lysis buffer) **: p<0.01 compared to non-treated cells (N=3). (B): Electrophysiological recordings of Kv1.3 currents: current-voltage curves obtained with basal current (blue), after addition of 1 µM reduced Tb11a (Tb11a$_{red}$, light blue) and 4-AP (green); Error bars represent SEM; *n*=5.

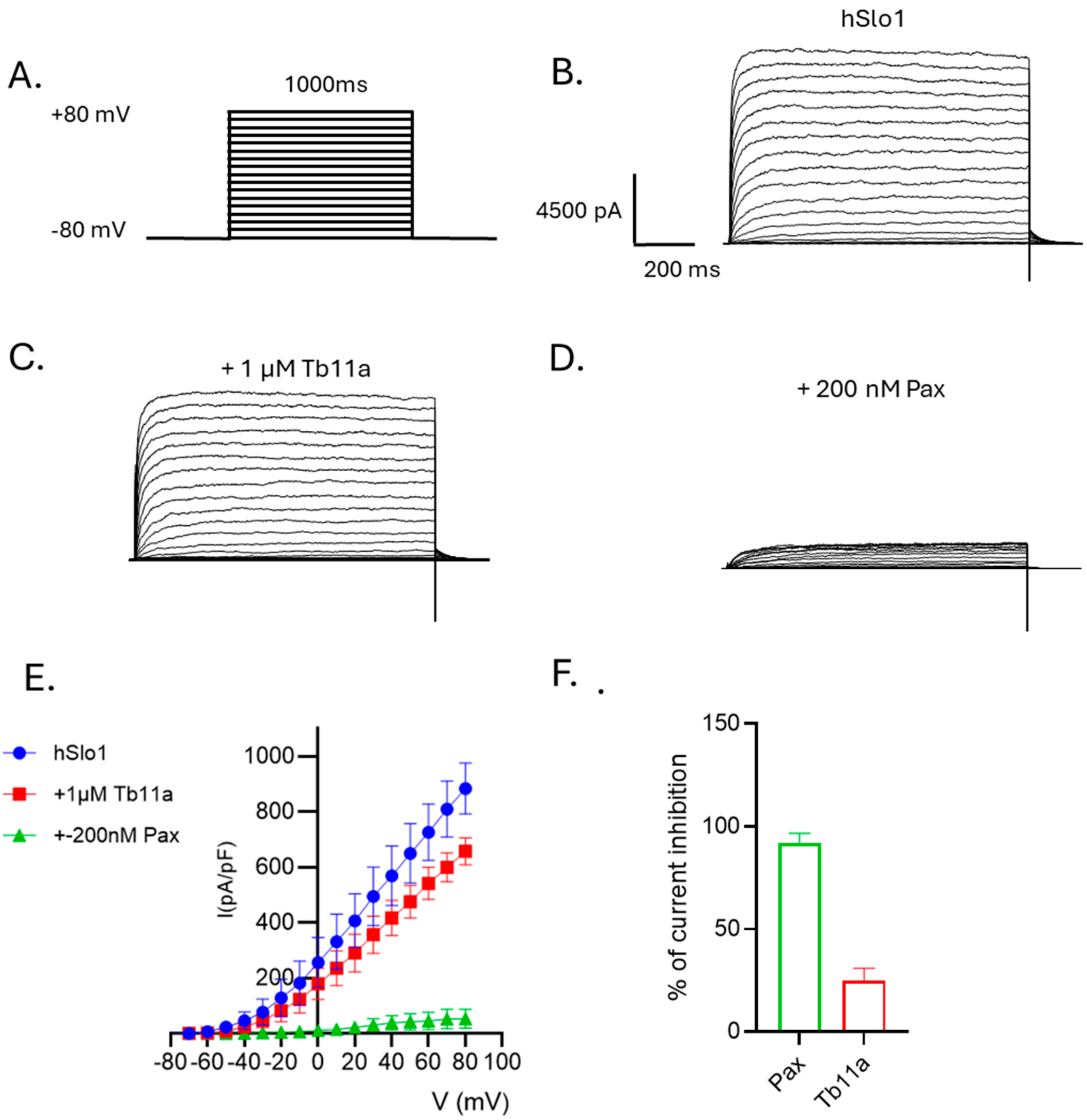

Figure S3: Effect of Tb11a on KCa1.1 currents. (A) Stimulation protocol for electrophysiological measurements. (B) Representative traces obtained with hSlo1 transfected cells. (B, C & D) Representative traces obtained from hSlo1 transfected cells on basal condition (B), after 1 µM Tb11a application (C) or after addition of 200nM paxilline (D). (E) Current-voltage curves obtained with basal current (blue), after addition of 1µM Tb11a (red) and 4-AP (green) Percentage of current inhibition at +80 mV upon treatment with 200nM- paxillin AP or 1 µM Tb11a (red) (Error bars represent SEM; *n*=3).

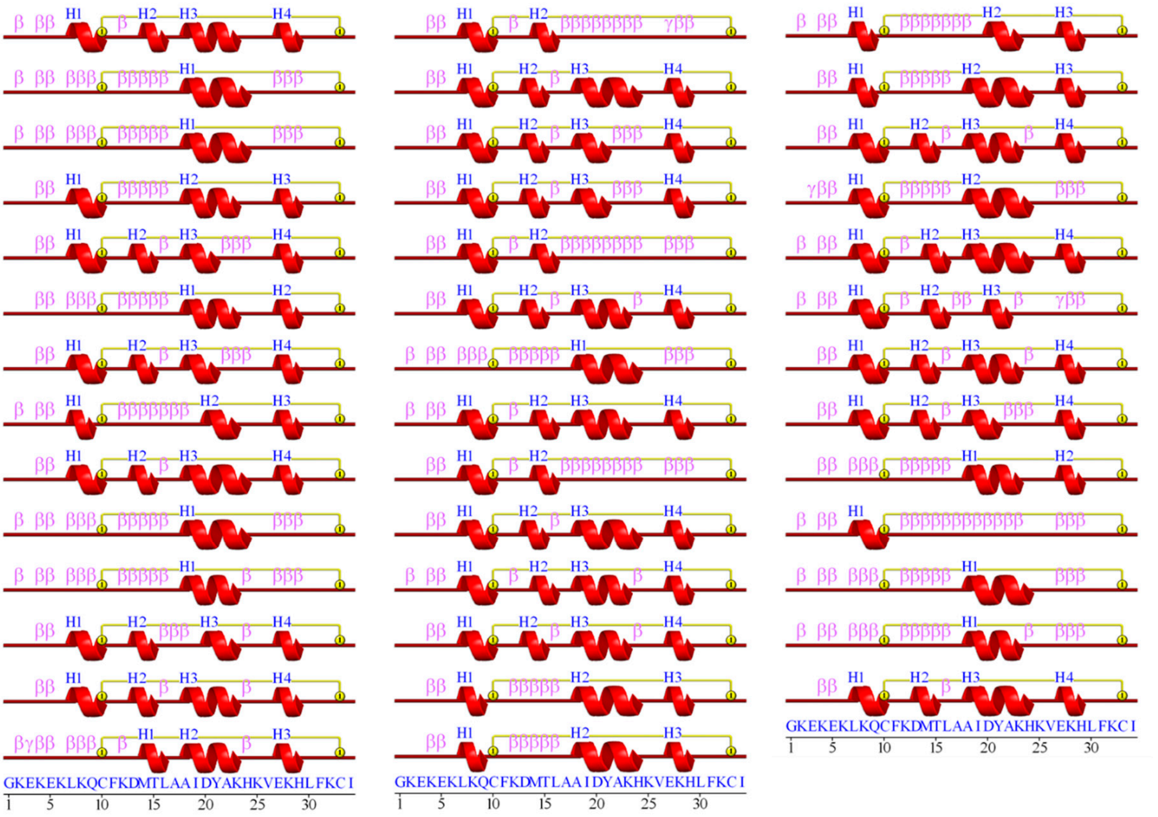

Figure S4: Secondary structure elements in the 42 selected models of Tb11a in complex with Kv1.3. The interaction between Tb11a and Kv1.3 was examined across the 42 selected models, revealing a consistent and well-organized network of contacts. The interface involves an average of 16 residues on Kv1.3 and 9 on Tb11a, with respective interface areas of (513 ± 40) Å² and (613 ± 40) Å². It is stabilized by 72 (± 10) non-bonded contacts per model, including 6 to 8 hydrogen bonds and a unique salt bridge (see Table S1 and Table S3).

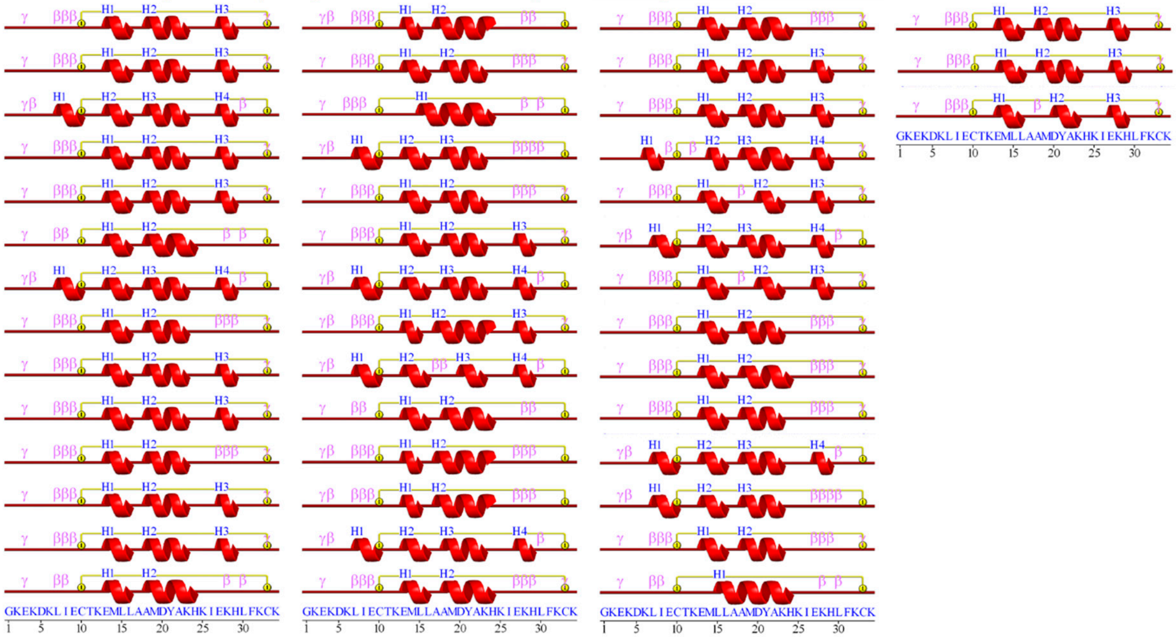

Figure S5: Secondary structure elements in the 45 selected models of Ta11a in complex with Kv1.3. The interaction between Ta11a and Kv1.3 was analyzed across the 45 selected models and demonstrated a well-defined and consistent interface, although less extended than that in the Tb11a-Kv1.3 complex. On average, Ta11a-Kv1.3interface consists of 11 residues on Kv1.3 and 4 on Ta11a, with interface areas of (295 ± 23) Å² and (366 ± 25) Å², respectively. Stabilization is provided by an average of 30 (± 5) non-bonded contacts per model, including 5 to 8 hydrogen bonds, but no salt bridges were detected (see Table S2 and S3).

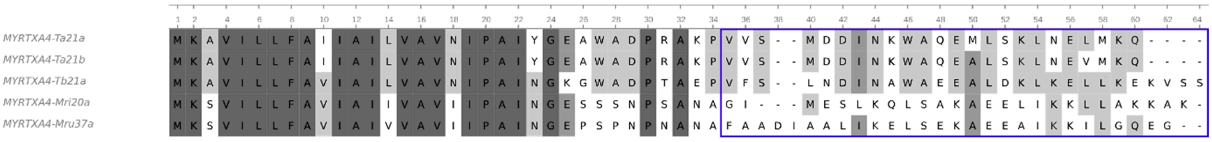

Figure S6: Alignment of precursors sequences of five venom peptides belonging to A4 family. Ta: Tetramorium africanum, Tb: Tetramorium bicarinatum, Mri: Manica rubida, Mru: Myrmica ruginodis. The part framed in blue corresponds to mature sequences.

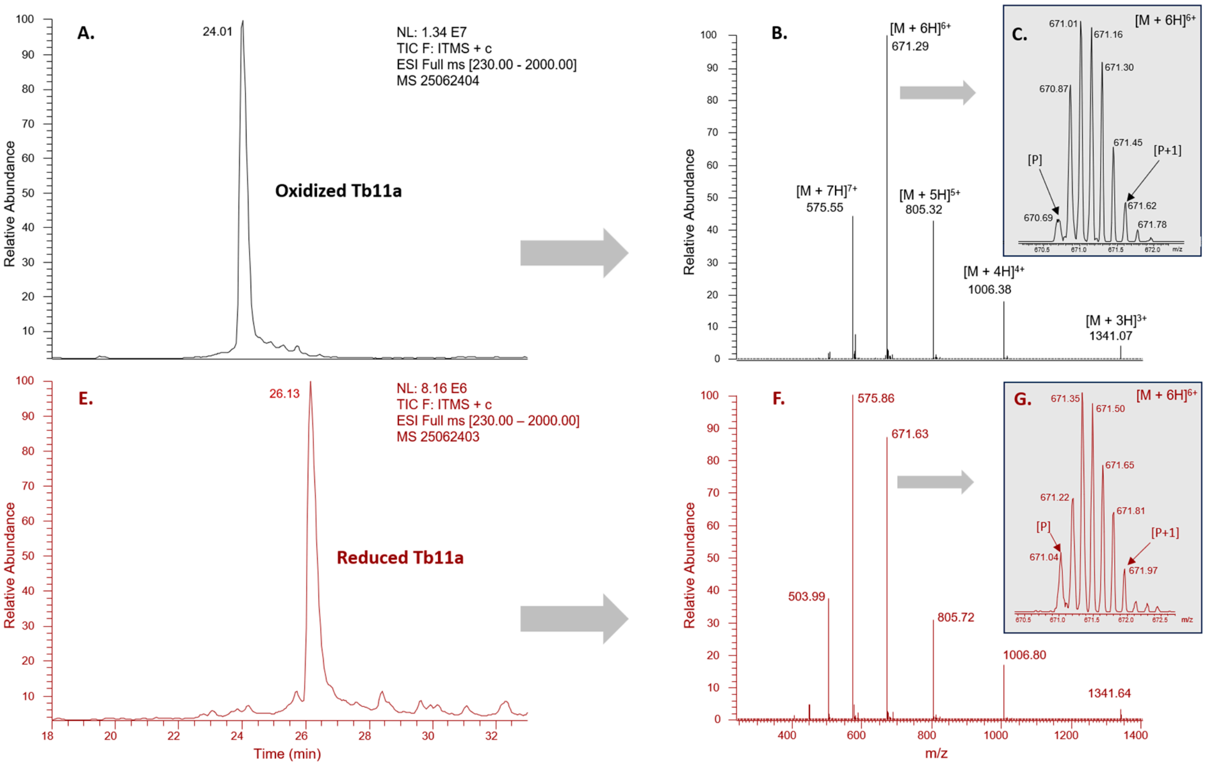

Figure S7: LC-ESI-MS analysis of oxidized and reduced Tb11a peptides. (A) and (E)**:** mass chromatogram with respective retention time of each peptide. (B) and (F): respective ESI-MS spectrum showing all states of charge. (C) and (G)**:** isotopic profile of $[M+6H]^{6+}$ used to calculate monoisotopic mass: ([P]x6)-6) result to 4018,14 for oxidized Tb11a, and 4020,24 for reduced Tb11a.

Table S1: Non-bonded contacts between Tb11a and Kv1.3 residues across selected Models.

Tb11a	Kv1.3	Number of non bounded contacts	Number of structures	% of structures	Mean number of non bound contacts
**D13**	(B)P424	106	33	78,6%	3
	(B)T425	67	22	52,4%	3
	(B)S426	424	41	97,6%	10
**M14**	(B)S426	11	4	9,5%	3
**L16**	(B)P424	21	20	47,6%	1
	(B)S426	2	2	4,8%	1
	(B)V453	2	2	4,8%	1
**A17**	(B)S426	22	22	52,4%	1
	(B)H451	38	38	90,5%	1
**Y21**	(C)Y447	215	41	97,6%	5
	(B)G448	267	41	97,6%	7
	(C)G448	229	42	100,0%	5
	(C)D449	177	42	100,0%	4
	(B)H451	89	39	92,9%	2
**H24**	(D)Y447	47	18	42,9%	3
	(C)G448	163	42	100,0%	4
	(D)D449	112	26	61,9%	4
	(C)H451	131	40	95,2%	3
**K25**	(A)Y447	120	39	92,9%	3
	(B)Y447	59	22	52,4%	3
	(C)Y447	23	11	26,2%	2
	(D)Y447	91	26	61,9%	4
	(A)G448	83	26	61,9%	3
	(C)G448	194	42	100,0%	5
**V26**	(A)D449	68	34	81,0%	2
**E27**	(A)G448	117	42	100,0%	3
	(A)H451	200	37	88,1%	5
**H29**	(A)S426	117	30	71,4%	4
	(A)D449	71	42	100,0%	2
	(A)H451	3	3	7,1%	1
**L30**	(A)H451	8	8	19,0%	1

Table S2: Non-bonded contacts between Ta11a and Kv1.3 residues across selected models.

Ta11a	Kv1.3	Number of non bounded contacts	Number of structures	% of structures	Mean number of non bound contacts
**A17**	(B)S426	2	2	4,4%	1
**Y21**	(B)G448	119	44	97,8%	3
	(B)D449	182	44	97,8%	4
	(B)H451	17	8	17,8%	2
**K23**	(B)S426	2	2	4,4%	1
**H24**	(C)G448	129	38	84,4%	3
	(C)D449	172	39	86,7%	4
	(C)H451	83	24	53,3%	3
	(D)G448	52	17	37,8%	3
	(D)D449	78	19	42,2%	4
**K25**	(A)Y447	129	45	100,0%	3
	(B)Y447	125	45	100,0%	3
	(C)Y447	127	45	100,0%	3
	(D)Y447	148	45	100,0%	3
	(D)G448	121	45	100,0%	3
**I26**	(D)D449	31	16	35,6%	2
	(D)H451	49	33	73,3%	1

Table S3: Comparison of Tb11a-Kv1.3 and Ta11a-Kv1.3 complexes interaction metrics. A third docking analysis was conducted between Ta11a and Kv1.3 in which the active residues were specified based on **Table S1**, reflecting interactions observed in the docking between Tb11a and Kv1.3. For Ta11a, the active residues included **E13, L16, A17, Y21, H24, K25, I26, E27, and H29.** For Kv1.3, the active residues were defined as P**424–S426** and T**443–H451 on the four subunits.** Passive residues for both partners were assigned automatically by HADDOCK. The entire peptide was modelled as fully flexible during the docking process. Residues D**422**–F**428 and** T441 to V453 were set as fully flexible on the four subunits for Kv1.3. Analysis of the generated structures was performed using the same criteria described in the main text.

	**Tb11a**	**Ta11a**	**Adjusted Ta11a**
Non-bonded contacts (avg ± SD)	72 ± 10	30 ± 5	50 ± 8
Hydrogen bonds (range)	6–8	5–8	7-9
Salt bridges (range)	1	None observed	0-1
Interface area - Kv1.3 (avg ± SD, Å²)	513 ± 40	295 ± 23	415 ± 26
Interface area - Toxin (avg ± SD, Å²)	613 ± 40	366 ± 25	506 ± 27
Number of interface residues - Kv1.3 (avg ± SD)	16 ± 1	11 ± 1	16 ± 1
Number of interface residues - Toxin (avg ± SD)	9 ± 1	4 ± 0	7 ± 1

Table S4: Non-bonded contacts between Ta11a and Kv1.3 residues across the selected models for the "adjusted" docking run.

Ta11a	Kv1.3	Number of non bounded contacts	Number of structures	% of structures	Mean number of non bound contacts
L16	(B)P424	38	11	52,4%	3
	(B)S426	22	11	52,4%	2
A17	(B)S426	39	19	90,5%	2
Y21	(B)S426	13	7	33,3%	2
	(B)G448	57	21	100,0%	3
	(B)D449	128	21	100,0%	6
	(B)M450	41	18	85,7%	2
	(B)H451	70	21	100,0%	3
H24	(C)G448	47	16	76,2%	3
	(C)D449	47	14	66,7%	3
	(C)H451	48	17	81,0%	3
	(D)G448	13	11	52,4%	1
K25	(A)Y447	36	21	100,0%	2
	(B)Y447	50	21	100,0%	2
	(C)Y447	44	21	100,0%	2
	(D)Y447	39	21	100,0%	2
	(A)G448	34	16	76,2%	2
	(D)G448	16	11	52,4%	1
I26	(A)S426	11	9	42,9%	1
	(A)G448	7	6	28,6%	1
	(A)D449	81	18	85,7%	5
	(A)H451	19	9	42,9%	2
E27	(A)H451	40	9	42,9%	4
H29	(A)S426	36	11	52,4%	3
